# Work Schedule Preferences and Shift Schedules of Per‐Diem Nurses: A Descriptive Cross‐Sectional Study of Online Labour Platform Data—Empirical Research Quantitative

**DOI:** 10.1002/nop2.70456

**Published:** 2026-02-22

**Authors:** Marcel Dettling, Florian Liberatore

**Affiliations:** ^1^ ZHAW School of Engineering Institute of Data Analysis and Process Design Winterthur Switzerland; ^2^ ZHAW School of Management and Law Winterthur Institute of Health Economics Winterthur Switzerland

**Keywords:** online labour platforms, per‐diem nurses, per‐diem nurse work, shift work, work preferences

## Abstract

**Aims:**

The paper analyses the work schedule preferences and actual shift work schedules of per‐diem nurses using data from an online labour platform that allows institutions to book per‐diem nurses for single shifts.

**Design:**

Descriptive cross‐sectional study of data from a Swiss online labour platform.

**Data Sources:**

Comprehensive data from a Swiss online labour platform about shift availabilities and resulting shift work for the booking period of 2022 (full sample). On the platform, healthcare providers can book per‐diem nurses for single shifts based on the nurses' individual shift availability offers.

**Results:**

The distribution of the 2022 shift availability count per nurse has a median of 15, resulting in 12 shifts worked, with higher success rates for nurses offering more availabilities. This corresponds to a global conversion rate of 87.95%. On average, per‐diem nurses are booked by three different institutions. The median time between entry of a shift availability and booking by an institution is 7.3 days, whereas the time between the booking date and the shift is on average 24.1 days.

**Conclusion:**

Per‐diem nurses on online labour platforms are booked in advance rather than on a short‐term basis. The low number of shifts offered and worked as a per‐diem nurse reveals that per‐diem nurse work is more of a side job than a substitute for a permanent position. The low number of annual shifts per institution highlights the potential issue in per‐diem nurse work of a lack of familiarity with institutional processes and structures.

**Implications for Nursing Management:**

The average lead time of 24.1 days on the platform indicates that shift managers should consider the use of per‐diem nurses according to expected shift demands during shift planning instead of using them on a short‐term basis. The low number of shifts worked as a per‐diem nurse per institution confirms the challenges that temporary nurses face as a result of their low institution‐specific work experience.

**Impact:**

What problem did the study address? The quality of the working experience as a per‐diem nurse, as well as the effectiveness of the deployment of these nurses in institutions, depends to a great extent on the patterns of work schedule preferences and the actual implementation in real work schedules in the institutions. Data from online labour platforms allow a comprehensive analysis of these patterns revealed by shift availabilities of temporary nurses and resulting patterns of actual shift work schedules based on booking information. What were the main findings? (1) Institutions book per‐diem nurses more on a monthly than on a short‐term basis. (2) The low number of shift availabilities annually and resulting annual shift volume reveal that per‐diem nurse work is more of a side job than a substitute for a permanent position. (3) Per‐diem nurses offer and are booked for a variety of shift types and not only for certain ‘preferable/unpreferable’ shifts. (4) On average, per‐diem nurses work in an individual institution for only a low single‐digit number of shifts. Where and on whom will the research have an impact? Nursing managers will learn about relevant statistics and their relevance in using online labour platforms for their shift planning. Nursing researchers will learn about the potential online labour platform data offer for analysing the characteristics, volume and patterns of per‐diem nurse work.

**Patient or Public Contribution:**

Per‐diem nurses were involved solely in the data provision process, by giving their consent to the analysis of the platform data. No participant contributions were required for the study's design, data analysis or interpretation.

**Reporting Method:**

I state that I have adhered to relevant EQUATOR guidelines (STROBE checklist).

## 1 Introduction

Worldwide, healthcare institutions increasingly staff shifts with temporary nurses, also referred to as agency nurses or travel nurses (Senek et al. 2020; Djukanovic et al. 2023). A sub‐category of temporary nurses is per‐diem nurses, who can be deployed for single shifts or days by healthcare institutions (Gan 2020). Institutions use them either to cover short‐term absences (Senek et al. 2020) or for flexible staffing models, depending on fluctuations in demand (Ejebu et al. 2021). Per‐diem staffing reflects labour market flexibility by enabling institutions to adapt to fluctuating demand through temporary workforce arrangements (Atkinson 1984). Per‐diem nurse work is a controversial issue because of the short‐term deployment, which has mixed outcomes for both the per‐diem nurses themselves and the deploying institutions (Aiken et al. 2007). According to dual labour market theory (Doeringer and Piore 1985), per‐diem nursing occupies a secondary labour market position, characterised by lower institutional integration, limited advancement and job precarity.

The valence of nurse‐related and institution‐related outcomes of per‐diem nurse work depends on the interplay between availability of per‐diem nurses and deployment decisions by institutions (Manias et al. 2003; Wynendaele et al. 2021). The quality of the working experience as a per‐diem nurse depends on the extent to which their working preferences are met in actual work schedules in the deploying institutions, whereas the effective deployment of these nurses in an institution depends on the patterns of work schedule preferences as well as the work schedules of the temporary nurses across institutions.

The emergence of online labour platforms (Meijerink and Arets 2021) for hiring temporary nurses offers a valuable new secondary data source that can provide a greater understanding of the work preferences of temporary nurses based on the binding shift availability offers and shift work characteristics documented by the bookings (Ahmadi Shad et al. 2025). On these platforms, temporary nurses offer shift availabilities that can be booked by institutions; this reveals their work preferences. A shift availability is a binding offer posted on the platform by a nurse indicating that he/she is available for a certain shift on a certain day (date); these can then be booked by healthcare institutions. A booking is a binding agreement that the per‐diem nurse will work on a certain day for exactly one shift in the institution that has booked the availability on the online platform. This provides details about the actual work schedules of these nurses. All activities (availability entries, bookings and planned shift date) are stored on the servers of these platforms and are therefore available for analysis. Per‐diem nurse work via online platforms aligns with core features of the gig economy, including platform‐mediated matching, project‐based engagement and high worker autonomy in choosing assignments (De Stefano 2016; Meijerink and Keegan 2019).

## 2 Background

There exists research on the topic of work preferences of temporary and the sub‐category of per‐diem nurses as well as on institutional development decisions of temporary nurses. *Regarding work preferences* of per‐diem nurses, the main motivations for engaging in work as a per‐diem nurse are the burden of occupation and working conditions (Birmingham et al. 2019; Hult et al. 2022), flexibility, less involvement in office politics (Simpson and Simpson 2019), work–life balance while earning supplemental income (Gan 2020), working closer to the patients (Berg Jansson and Engström 2017; Djukanovic et al. 2023), and more task‐oriented work (Gan 2020) and opportunities for inter‐organisational workplace learning (Berg Jansson and Engström 2022). Studies have shown that self‐scheduling, which is the primary characteristic of a per‐diem work arrangement, can positively influence nurse job satisfaction and work–life balance (Wynendaele et al. 2021), enabling nurses to better align their work schedules with their personal needs and preferences.

The extent to which their preferences are met and the associated positive outcomes for nurses are achieved depends on the number and types of shifts for which the institution books them (Wynendaele et al. 2021). Previous studies have found detrimental results for temporary nurse schedules and associated negative outcomes for nurses. Last‐minute shift notifications, occasional cancellations and ward assignments often caused anxiety and frustration and led to financial and logistical difficulties (Manias et al. 2003). Additionally, nurses sometimes faced inappropriate ward allocations, with some being pressured to work in settings outside their competence, causing stress and conflicts with permanent staff (Manias et al. 2003). Furthermore, temporary work has been found to be associated with working a lot of overtime, double shifts and inconvenient working hours (Epstein et al. 2023). De Cordova et al. (2013) found that it was mainly night shifts being covered by temporary staff types and that these sometimes were not the nurses' shift preferences. Hence, the extent to which nurses' preferences are met by actual shift work patterns has been identified as an important area for future research (Ejebu et al. 2021; Epstein et al. 2023).

Working in different institutions with different workflows for single shifts has been associated with difficulties finding the workplace and a lack of orientation to the work environment, which in turn lowers effectiveness and efficiency of the work as a per‐diem nurse (Simpson and Simpson 2019). Furthermore, it may limit the motivation to work closer to the patients. Based on semi‐structured interviews among nurse managers, Gan (2020) infers that nurses in a per‐diem working arrangement prefer deployment in a low number of institutions in order to have consistent work environments. At the same time, temporary nurses may benefit from the work experience in different institutions. Therefore, the extent to which these challenges and opportunities of varying workplaces materialise depends on the number of different institutions that book the per‐diem nurse.

*From the perspective of a deploying institution*, per‐diem nurses are a supplemental source for staffing demands (Kortbeek et al. 2015). However, the extent to which vacant shift demands are covered depends on the availabilities of the nurses for certain shift types (Goodman‐Bacon and Ono 2007) and how institutions can allocate permanent and internal float pool nurses along with per‐diem nurses (Manias et al. 2003). Temporary nurses are criticised for cherry‐picking available shifts, in that they may only offer availability for favourable shifts or only take favourable shifts, leaving permanent nurses to cover the remaining shift demands (Lien 2022). By contrast, a review by O'Connell et al. (2024) revealed predominantly positive organisational outcomes of electronic self‐rostering systems, allowing nurses to select their preferred shifts for working within a schedule period; however, they also identified the potential of overtime and requests for shift changes as detrimental outcomes. The extent to which temporary nurses must be deployed further depends on roster approval lead time for the permanent staff, with shorter lead times resulting in more temporary nurse use (Drake 2018).

Moreover, the deployment of temporary nurses is associated with potential negative effects on efficiency, effectiveness (Aiken et al. 2007) and quality of care (Manias et al. 2003). Negative effects on nurse and patient safety outcomes have been identified by studies, such as Bae et al. (2010) and Hart and Davis (2011). The deployment of temporary nurses leads to additional work responsibilities of permanent nurses, such as onboarding and supervising temporary nurses, which adds to their existing workload and subsequent outcomes (Gahrmann and Klumb 2024). There are also studies showing that per‐diem nurse staffing has no effect on quality of care (Hurst and Smith 2011; Bae et al. 2015).

Despite the potential lack of commitment of the per‐diem nurse towards the institution and lower motivation to achieve good work outcomes, the extent and valence of these negative effects mainly depend on the familiarity with institutional processes and protocols, data access and support. Familiarity with an institution and its work environment depends on the frequency of shifts a per‐diem nurse works in the same institution.

Given these dependencies between work preferences of per‐diem nurses and deployment decisions by institutions, research on per‐diem nurse work lacks findings from secondary data about the patterns of shift work schedule preferences and actual work schedules of per‐diem nurses. As noted earlier, existing studies mainly rely on findings from qualitative studies and survey data, and base themselves on information about stated motivations for temporary work and work experiences. Furthermore, staffing data are limited to single institutions, which does not allow analysing per‐diem nurse deployments across institutions, with the exception of the e‐rostering data from the NHS examined in Drake (2018).

This study is among the first to use data from online labour platforms, analysing the work schedule preferences revealed and the related work schedules of per‐diem nurses based on a single data source. The data help to understand the shift preferences of per‐diem nurses and how they are met by actual bookings by different institutions on the platform. It also contributes to research on per‐diem nurse staffing by healthcare institutions, revealing how often healthcare institutions book per‐diem nurses, for which shifts, and in which time frame. Moreover, it provides data on varying workplaces and work experiences in single institutions, contributing to the discussion on workplace familiarity of per‐diem nurses.

The aim of this study was to analyse the work schedule preferences and actual shift work schedules of temporary nurses using shift availability and booking data from a Swiss online labour platform for temporary nurses for the Year 2022. This study examines the following research questions:

1. What are the work schedule preferences of temporary nurses regarding preferred workloads and shift types?
2. What are the conversion rates of shift preferences, actual work schedules of temporary nurses regarding actual workloads, shift types, varying workplaces and number of shifts per institution?
3. What is the temporal perspective of per‐diem nurse work regarding pre‐planning preferences, offering periods and lead times?

## 3 Methods

### 3.1 Study Design and Setting

We conducted a descriptive cross‐sectional study of data from a Swiss online labour platform for the Year 2022. In 2022, the platform had 132 Swiss healthcare institutions (hospitals, nursing homes, home care, ambulatory centres) as clients for shift‐based bookings of temporary nurses. A total of 309 per‐diem nurses were employed by the temporary agency offering shift‐based per‐diem nurse work on the platform. They are paid a fixed hourly rate by the agency according to the number of shifts they take. The temporary agency also pays social security contributions for the employed temporary nurses. Booking institutions as customers of the agency must pay a fee per shift for the intermediation service. The rationale for choosing annual data from 2022, although data from earlier years were available, was that between 2016 and 2020 a smaller number of temporary nurses and clients were active on the platform and booking patterns in the period from 2020 to 2021 were affected by the COVID‐19 pandemic. Therefore, we chose to extract the data from 2022 from the platform for the study. Additionally, having an annual perspective on work schedule preferences and actual work schedules facilitates the interpretation of the absolute number because of the seasonal fluctuation of staffing numbers.

On the platform, the per‐diem nurse work booking process is as follows: temporary nurses must enter their shift availabilities. Such a shift availability offers signals the capacity to take over a shift on a given calendar day. Availabilities can comprise a single window (e.g., morning shift), or up to four windows (e.g., morning, day, evening and night).

Institutions can then book nurses by choosing an availability for one single window per calendar day. Per‐diem nurses can exclude institutions that they do not want to work in; in that case, their shift availabilities will not be visible to those institutions. CVs of the nurses are uploaded, so booking organisations can inform themselves about qualifications and experience. The booking of a shift availability by an institution leads to a binding agreement between the institution and the temporary nurse. The matching (an assignment being offered and accepted) may take place as late as 24 h before the start of the shift in question. The contracting, coordination and communication of a work assignment happens over the platform in near‐real time with no delay.

### 3.2 Sample

Our study is based on the full sample of temporary nurses who were (1) employed by the temporary agency for shift‐based per‐diem nurse work, (2) registered on the platform and (3) had placed at least one shift availability on the platform in 2022. All employees of the temporary agency were informed by letter about the study and could decline to give their consent to the use of their individual data. With this informed consent process, all temporary nurses agreed to participate in the study. This gave us a total of 309 nurses, with a median age of 38.6 years (28.7–60.8 years for the 10% and 90% quantiles), out of which 74.76% were female and 96.12% were of Swiss nationality. The institutions represented were a total of *n* = 132 Swiss healthcare provider customers of the temporary agency. They include 33 large, 17 basic and 24 specialised hospitals, 14 home care units, 33 nursing homes and 10 other institutions.

### 3.3 Data and Variables

**FIGURE 1:**
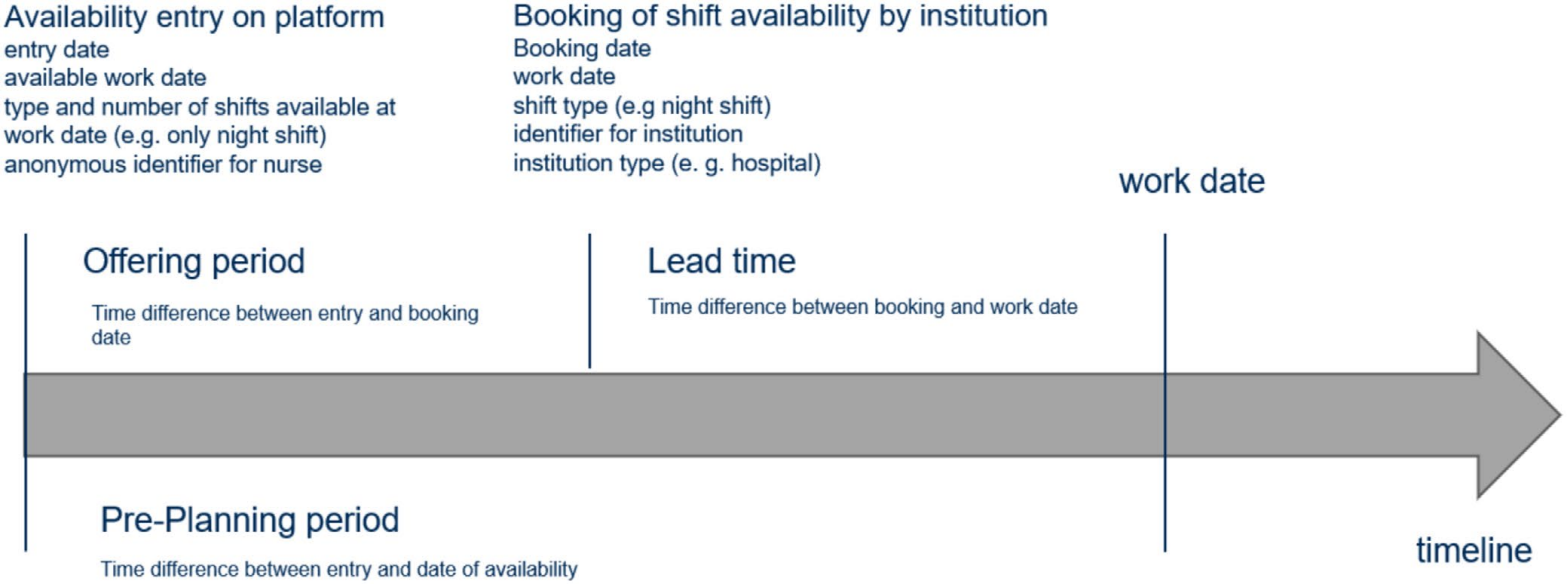
Overview of booking logic of the platform and available data variables.

The platform data were provided on an Excel sheet on which the nurses' names were replaced with a unique, anonymous identifier. The observation unit (row) is an availability of a per‐diem nurse to work on a specific day. These shift availabilities are characterised by several variables (columns) with the date, the shifts offered and (if applicable) the institution that booked the labour. No data processing regarding missing values, duplicate entries and outliers were needed. Figure 1 displays the booking logic as well as the available variables in the platform data. Each entry of a shift availability on the platform comprises the entry date, available working date, type and number of shift types available on the working date, and an identifier for the nurse who placed the entry. Due to the characteristics of the platform, per‐diem nurses may enter from one (e.g., only night shift) up to four shift availabilities (early, middle, late and night shift) for a specific day. Once an institution books a shift availability, the data about the entry is enriched with information about the booking date, the chosen window and an identifier for the institution, as well as the institution type (hospital etc.) of the booking institution. In addition to the available variables, the time in full days between entry and date of availability as proxy for the preferred pre‐planning time, as well as the lead time (calculated as time difference in days between booking and working date), were calculated as new variables. This is shown in Figure 1.

### 3.4 Statistical Analysis

All subsequent computations were done with R for Windows (version 4.2.2, R Core Team, 2022). The plots shown in this paper were produced using the ‘ggplot2’ package. Most of the analyses presented here are based on nurse level, reporting their participation, pre‐planning behaviour, etc. This means that the nurse‐specific distribution of aspects, such as the times between entry of an availability in the platform and the shift itself had to be condensed into a representative number. These representants are then either visualised or boiled down to a number that represents the behaviour of the different nurses. Because most of the distributions involved are right‐skewed, all summary statistics are presented as medians rather than means. To provide some insights into the dispersion of the values, we report the 10% and 90% quantiles (rather than the standard deviations). These were computed using the quantile() function in R.

## 4 Results

### 4.1 Work Schedule Preferences of Per‐Diem Nurses

#### 4.1.1 Shift Volume Preference

**FIGURE 2:**
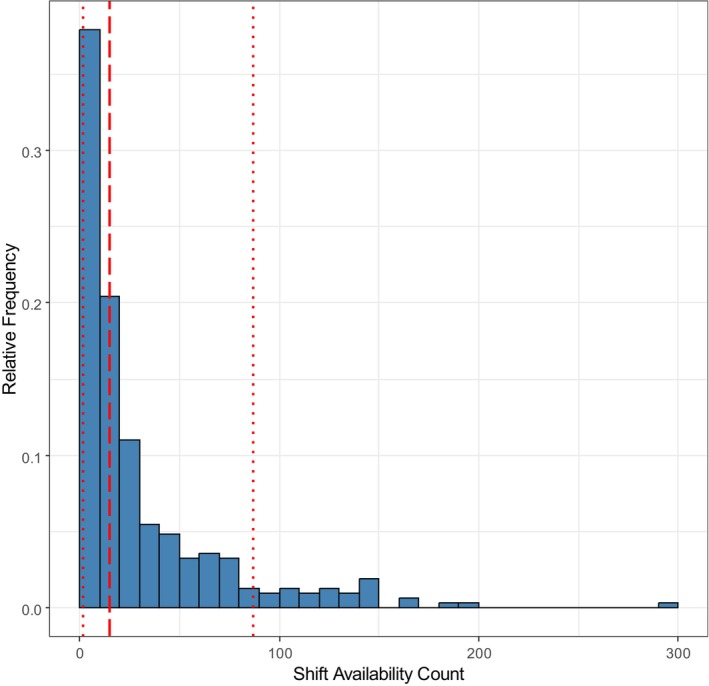
Distribution of availability frequency of per‐diem nurses in 2022.

First, the number of shift availabilities was assessed as a proxy for the workload preference of the per‐diem nurses on the platform. Each of the 309 nurses in total offered a certain number of shift availabilities in 2022. The distribution of that number is shown in Figure 2. It has a median of 15 shift availabilities per per‐diem nurse (highlighted by the dashed line) and a big dispersion, with 10% and 90% quantiles of 2 and 87 shift availabilities per per‐diem nurse (shown by the dotted lines).

#### 4.1.2 Shift Type Preferences

**FIGURE 3:**
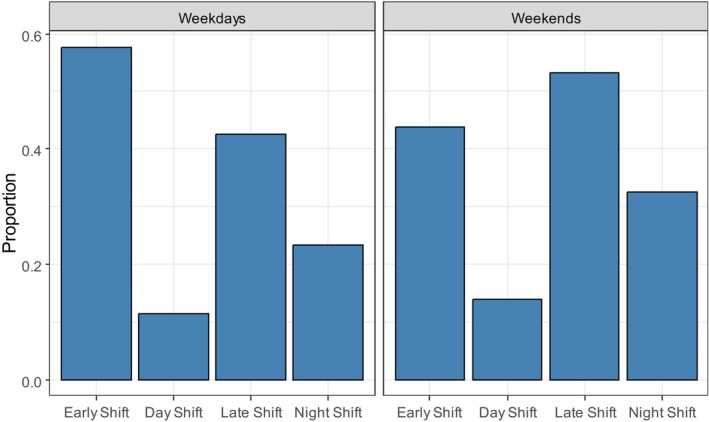
Proportion of availabilities that include the offer of specific shift types.

The nurses' preferences for certain days of the week (week vs. weekend; Monday to Sunday) and shift type on a day (early, middle, late and night shifts) are shown in Figure 3, which also shows the fraction of shift availabilities of the per‐diem nurses on the platform that included the early, middle, late and night shifts. For this analysis, we distinguished between weekdays and weekends. The fractions reported add up to a value > 1 because more than one shift can be offered in a shift availability. The readiness to work in the day shift exhibits low values: only around 11% (weekdays) to 13% (weekends) of nurses offered that shift in their availabilities. Apart from that, the offerings in the shift availabilities reveal a higher preference for the early shift on weekdays as opposed to weekends, whereas the proportions for the late and night shifts are higher for weekends. But even on weekends, an abundant number of early shifts are offered, as are late and night shifts on weekdays.

**FIGURE 4:**
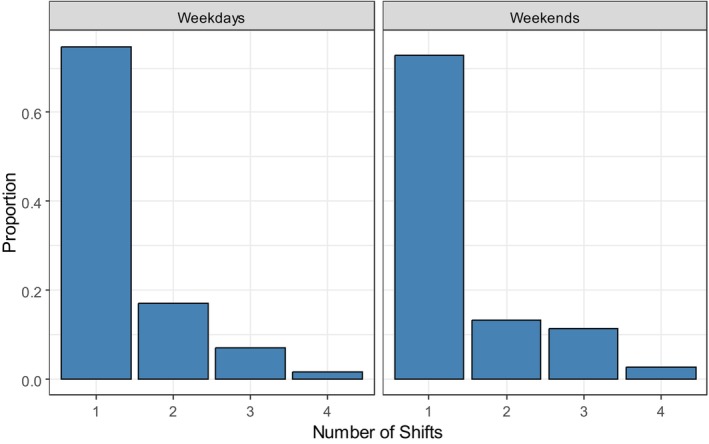
Nurses' offerings of different shift types per working date in 2022.

Figure 4 shows the results of the analysis of flexibility in preferences for shift types on weekdays and weekends. The results reveal that only a single shift was offered in around 75% of the shift availabilities (e.g., only the early shift on the available working date), with the difference between weekdays and weekends being small. For the remaining 25%, it was generally two or three shifts that were offered, with all four being offered only very rarely. There was a slight tendency to offer more shifts per availability on weekends than on weekdays, with the respective means being 1.35 shifts (weekdays) and 1.43 shifts (weekends).

### 4.2 Conversion Rates of Shift Preferences and Work Schedule Characteristics of Per‐Diem Nurses

The second research question of the study addresses the conversion rates of shift preferences and subsequent characteristics of actual work schedules (number of work assignments, varying workplaces, number of shifts per institution and shift types) of per‐diem nurses, which are revealed by the booked single shift work assignments on the platform. A booking is made when an institution books a temporary nurse's offer of availability. Therefore, analysis of the platform data reveals the conversion or success rate of shift availability offers based on the proportion of shift availabilities that were booked by institutions.

#### 4.2.1 Conversion Rates of Shift Availabilities

**FIGURE 5:**
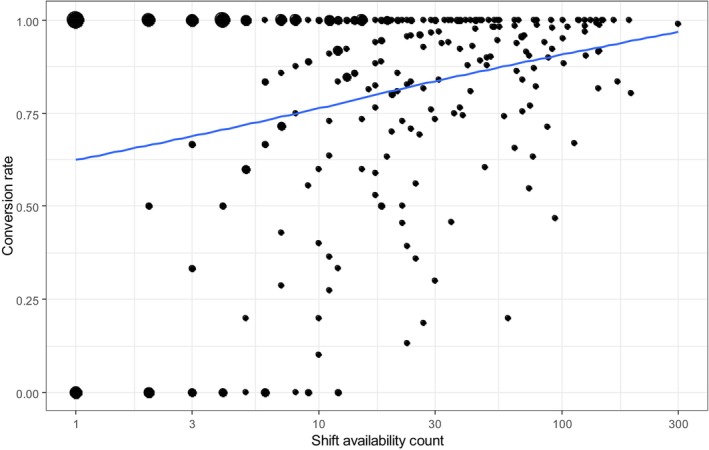
Conversion rates from availabilities to bookings, depending on the number of availabilities of a nurse.

The conversion rate indicates the extent to which shift availability offers led to actual bookings and subsequent deployments. Of the 9783 shift availabilities of the per‐diem nurses in total, 8604 were booked and resulted in a work assignment, resulting in a global conversion rate of 87.95%. However, the individual variation between per‐diem nurses is substantial. We created a scatterplot displaying the correlation between number of availabilities and conversion rates; this is shown in Figure 5. The size of the data points corresponds to the number of nurses in the respective configuration. The x‐axis is logarithmic. The scatterplot smoother indicates that the conversion rate increases with the number of availabilities. The results show that a total of 27 nurses (with up to 12 availabilities entered in the platform) were without success, but a substantial number, 130 nurses, had all their offers converted into work assignments. The scatterplot smoother clearly shows a correlation between number of availabilities of a nurse and the conversion rate of the availabilities.

#### 4.2.2 Actual Workloads

A total of 282 nurses (out of 309) had at least one of their work offers successfully converted. The distribution of the number of work assignments is strongly right‐skewed and very similar in appearance to the distribution of work offers shown in Figure 2. The median is a value of 12 worked shifts annually per per‐diem nurse. Here again, the distribution is huge, with the 10% and 90% quantiles of the distribution ranging from 1 to 87 shifts worked annually per per‐diem nurse.

#### 4.2.3 Varying Workplaces

**FIGURE 6:**
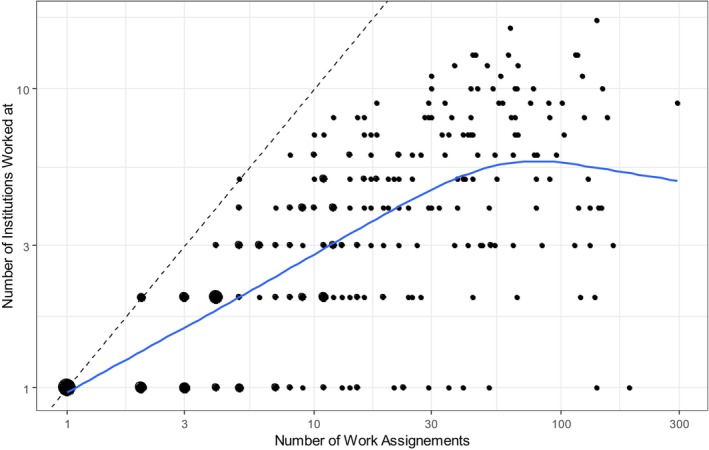
Dispersion of workplaces of a per‐diem nurse in relation to the number of work assignments.

In considering the second research question, we analysed the dispersion of workplaces that nurses offering their availability on the platform face. A total of 133 institutions booked nurses. Across the 282 per‐diem nurses that had at least one work assignment over the platform, the median number of institutions in which they worked in 2022 was 3, with a range between 1 and 9 different institutions per year in the 10% and 90% quantiles. As the number of different workplaces may vary with the number of annual shifts per per‐diem nurse, we charted this on a scatterplot (see Figure 6) that shows the number of institutions worked at in relation to the number of work assignments the nurses had. Both axes are logarithmic, and the point size corresponds to the number of nurses in the respective configuration. Being on the bisectrix (dashed line) means being at a different institution for every work assignment. The scatterplot smoother shows a steady increase in the number of workplaces, flattening out to a value between 5 and 6 institutions for the most frequent users of the platform with 30 or more work assignments.

#### 4.2.4 Number of Shifts Per Institution

**FIGURE 7:**
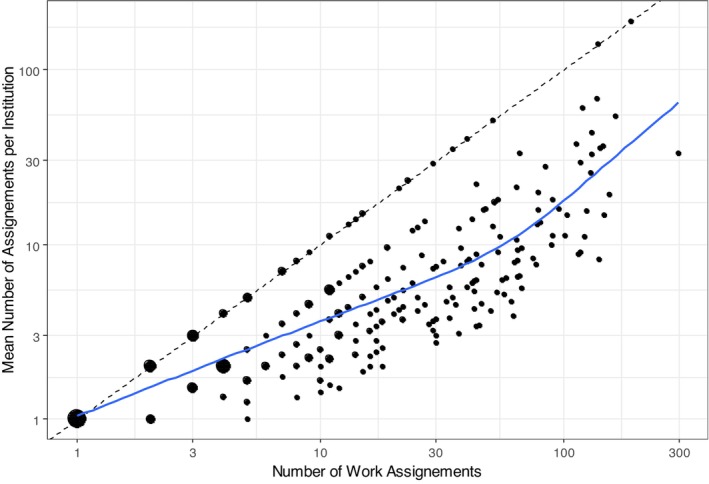
Mean number of times a nurse has worked in an institution, depending on the number of work assignments of the nurse, with a scatterplot smoother added.

To learn about the frequency of shift deployments in the same institution as an indicator of workplace familiarity, we computed the mean number of shifts worked per institution for each nurse. The distribution has a median of 4.1 with [1.3, 17.8] as the 10% and 90% quantiles. Again, the number of shifts per institution may vary with the number of annual shifts per per‐diem nurse. Here again, we created a scatterplot, which is shown in Figure 7. Both axes are logarithmic, and the point size corresponds to the number of nurses in the respective configuration. The data points on the bisectrix (dotted line) are per‐diem nurses who had all their assignments in just one institution. Nurses who worked just once at each institution are located on a horizontal line at *y* = 1. The number of assignments per institution has a weaker than linear increase by the number of work arrangements over the platform.

#### 4.2.5 Application of Shift Type Preferences in Work Schedules

**FIGURE 8:**
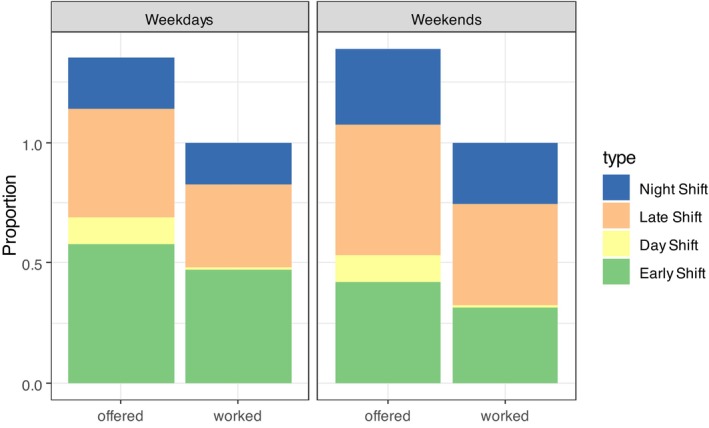
Shift preferences of nurses, compared with the distribution of how shifts were worked.

To understand how shift type preferences of per‐diem nurses are actually applied in the booking of these shift types, we compared nurses' shift preferences to the actual shift work schedules. As already mentioned, shift availability comprises either one, two, three or even four shift types (early, day, late and, night). However, nurses are only booked for a single shift on any given day. In Figure 8, we limit ourselves to successful platform entries that resulted in a work assignment and show the proportion of shift types offered and worked. Because a shift availability can contain more than one shift type per day, these proportions add up to a value exceeding 1. For the shifts worked, however, the sum is exactly 1.

Figure 8 shows that when nurses offer to work a day shift, this almost never materialises, because only very few providers do actually run a day shift. Further, a slightly higher prevalence of the early versus late/night shifts on weekdays is observed, whereas the opposite is seen on weekends. This transfers to shifts worked. Overall, except for the day shift, there seems to be little discrepancy between the shift types offered and shift types actually worked.

### 4.3 Temporal Perspective on the Rostering Process of the Per‐Diem Nurses on the Online Labour Platform

The third research question addresses the temporal sequence between shift availability entries, bookings and working dates, the pre‐planning preferences of per‐diem nurses, and how they are actually implemented in bookings by institutions.

#### 4.3.1 Pre‐Planning Period

**FIGURE 9:**
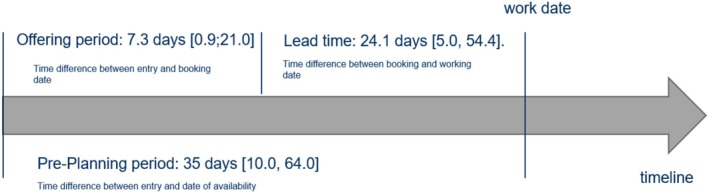
Overview of median of offering periods, lead times and overall pre‐planning periods (in days) of per‐diem nurses on the platform.

The temporal perspective comprises the pre‐planning behaviour of nurses, which is determined by analysing the time in days between the entry of the availability in the platform and the day the work takes place (pre‐planning period) (see Figure 9). This measure reveals preferences regarding the pre‐planning of potential work schedules. There is a distribution of the pre‐planning period for each per‐diem nurse, which is summarised by the mean. The median of this distribution across nurses was 35 days [10.0, 64.0].

#### 4.3.2 Offering Period

The offering period (the time between the entry date and the booking date) indicates how long a nurse must deal with uncertainty regarding the success of their shift availability offers on the platform. The median offering period is 7.3 days [0.9; 21.0] (see Figure 9).

#### 4.3.3 Lead Time

The lead time (the period between booking and working date) indicates the extent to which the per‐diem nurses are booked by institutions at short notice. Only around 15% of bookings happened at short notice (defined as 7 days or less in advance of the service), and that only in cases where nurses made themselves available at short notice. In more than half of cases, nurses had 3 weeks or more of lead time before their work. This is counterevidence to the claim that per‐diem nurses must be ‘on duty’ continuously. The median of the distribution is 24.1 days [5.0, 54.4] (see Figure 9).

## 5 Discussion

### 5.1 Summary and Contributions to Previous Literature

Our findings also have important theoretical implications for the field. The study is the first analysing self‐rostering behaviour of nurses and nurse scheduling in the setting of online labour platforms as labour markets for temporary work characterised by greater transparency and low transaction costs.

#### 5.1.1 Shift Volume/Patterns

The results of the study offer quantitative data about the shift volume (preferred/actual) and shift types (preferred/actual) of per‐diem nurses based on preferences and actual work schedules across institutions revealed in the data. The offered annual shift volume of the nurses (preferred 15 annual shift availabilities as median; 12 actual annual shift bookings as median) on the platform indicates that the nurses offering temporary work on the online labour platform have rather low workloads in this work arrangement. Only a small number of the registered nurses on the platform worked full‐time as per‐diem nurses. This result underscores the findings of Gan (2020) that per‐diem nursing is a part‐time work arrangement as a source of supplemental income. Therefore, the shift preferences reflect availabilities that are compatible with personal obligations and schedules of potential permanent positions rather than preferences for working as a per‐diem nurse full‐time.

The shift availability patterns of per‐diem nurses depict a variety of preferred shift types (day, afternoon, night shift; week vs. weekend) across all per‐diem nurses. No particular preference for certain shifts is visible. The predominant reason for the lower number of availabilities of day shifts is that these shifts are very rarely booked because most institutions have a three‐shift model (early, late and night). It can be assumed that across the pool of per‐diem nurses, the diverse shift preferences and in turn, shift availabilities on the platform lead to a balanced distribution across all shift types. This is in line with the results of the scoping review by Ejebu (2021), which shows that shift type preferences vary according to nurses' personal characteristics and circumstances. However, for around 75% of the shift availabilities only one shift was offered within the day, which demonstrates a clear preference for a specific shift on each day. The small difference between offered shift availabilities and actual worked shifts, resulting in a match rate of 87.95%, shows that the per‐diem nurses can achieve their preferred shift schedules in actual bookings. The results therefore contribute to the research gap in how nurses' preferences are met by actual shift work patterns (Ejebu et al. 2021; Epstein et al. 2023), and show that the main motivations for engaging in temporary nurse work, namely flexibility and work–life balance, can be fulfilled by the work arrangement (Gan 2020). However, the success rate for per‐diem nurses on the platform increases with the number of shift availabilities offered annually, which shows that institutional demands for certain shifts may at least partly determine the success of the self‐rostering of per‐diem nurses on the platforms. Furthermore, the high success rates may be the result of the per‐diem nurses adapting their availability offers to the institutional demands on the platform after lower match rates in previous periods.

The patterns of the actual worked shifts exhibit patterns similar to those of the shift availabilities. These findings contradict the observations of De Cordova et al. (2013) that temporary nurses are booked for unfavourable shifts, such as night shifts. The high match rates indicate that per‐diem nurses realise the level of earnings they want to achieve by their shift availability offers on the platform.

#### 5.1.2 Temporal Perspective on the Rostering Process of the Per‐Diem Nurses on the Online Labour Platform

This study is the first to take a detailed look at the pre‐planning preferences of per‐diem nurses based on preferences revealed on an online labour platform and the pre‐planning of healthcare institutions regarding the deployment of per‐diem nurses in shift schedules. The cascade of placing shift availabilities on the platform, being booked by an institution and working the shift reveals the preferences of the nurses regarding their pre‐planning and how they are met by the time of the booking. On average, per‐diem nurses place shift availabilities 35 days in advance and follow a personal monthly shift scheduling approach. Seven days later and 24 days before the shift starts, they have certainty about the bookings in their shift schedule, which is comparable to the nurse scheduling plans for permanent nurses. It seems that per‐diem nurses either adapt the monthly pre‐planning processes based on former or actual scheduling experiences in work conditions as permanent nurses, or the monthly cycle best aligns with their plans in private life. Although short‐term bookings up to only 24 h in advance are possible, the mean period per nurse between match and shift start shows that institutions prefer per‐diem nurses with a solid lead time over short‐term; only 15% of the bookings were short‐term (7 days or less before shift start). These findings contrast with those of previous studies in which temporary nurses reported experiencing short‐term shift assignments (Manias et al. 2003). Either these findings must be interpreted in the context of that study's setting, the labour market conditions of the Year 2002 in the Swiss healthcare market (which was characterised by a nursing personnel shortage), or they indicate that healthcare institutions are now considering per‐diem nurses as an additional workforce in their ongoing planning rather than as replacements for short‐term absences of permanent staff, indicating a longer lead time in the rostering of permanent staff (Drake 2018).

#### 5.1.3 Varying Workplaces/Number of Shifts Per Institution

With a median of three different institutions in which the per‐diem nurses registered on the platform worked in 2022, the data reveal that per‐diem nurses are employed for single shifts but only in a single‐digit number of different institutions. This result helps to understand the extent and valence of nurse‐related and institution‐related challenges associated with the familiarity of the employing institutions and predictability regarding their workplace (Galais and Moser 2009; Simpson and Simpson 2019). The low number of annual shifts per institution in the results highlights a potential issue in per‐diem nurse work: lack of familiarity with institutional processes and structures.

### 5.2 Comparison With Findings From Systems in USA and UK

In the USA, per‐diem nurses often operate as independent contractors or through staffing agencies, and their shift availability is frequently determined through gig platforms or direct agency communication (Manias et al. 2003; Lien 2022). They are frequently scheduled on short notice, often on the same day, particularly in acute care settings. (Lien 2022). This stands in contrast to the current findings, which show that per‐diem nurses are typically booked several weeks in advance.

In the United Kingdom, the NHS uses bank and agency nurses to address staffing gaps, supported by electronic rostering systems that facilitate both advance and last‐minute scheduling (Drake 2017). Recent trends point to an increase in planned deployments, partially aligning with the advance scheduling patterns reported in the current study (Massey et al. 2009). Moreover, temporary nurses in the United Kingdom often work across multiple institutions but may do so more frequently per facility than those described in this study, potentially due to differences in platform models, contractual norms or workforce demand (Runge et al. 2017).

### 5.3 Limitations and Implications for Future Research

The study is not without its limitations. The platform data originate from a commercial platform of a single Swiss per‐diem nurse agency. Although the data originate from a full population sample of per‐diem nurses of the platform in 2022 and the company has a high market share, the data do not provide a comprehensive overview of temporary work handled by online platforms in Switzerland. Multi‐centre studies with data from different platforms and countries are recommended for future research. Moreover, the population on the platform may not reflect the variety of per‐diem nurses regarding work preferences. Future studies should use more aggregate data from different platforms to account for different platform characteristics. As soon as national monitoring systems for per‐diem nurse work are established, studies could more comprehensively analyse nurse preferences, per‐diem nurse work demand, and actual volume and work schedules on the health system level. Furthermore, the platform is configured for the matchmaking process and has shortcomings in data classification according to international norms, completeness and data quality. The working preferences are analysed based on availabilities on the platform, but per‐diem nurses may have additional working contracts as permanent nurses or in different sectors. Therefore, the results do not present a full picture of the work schedule preferences. This limitation calls for mixed‐method studies combining platform data with data from surveys among per‐diem nurses to fully capture multiple working contracts. Survey data would also allow for a better understanding of the motivations and adaptive behaviours of per‐diem nurses that are reflected in the platform data. Surveys among shift managers responsible for bookings on these platforms could help understand the motivations behind the booking behaviours.

Regarding actual work schedules, the platform data only contain booking information. They do not indicate any possible changes, for example a per‐diem nurse calling in sick or cancelling in the period between booking date and working date. Therefore, the data may overstate the work schedules as well as the potential of per‐diem nurse work as a flexible resource avoiding understaffing. Future studies should account for this by enriching the data sets with later changes and should aim to match platform data with actual attendance records from institutions.

The data analysis did not include information about education, qualifications, professional experience, household income, etc. Some of this information was available in the CVs accessible on the platform as PDF‐attachments to the individual entries of the per‐diem nurses. The study consent did not allow analysing these data. Future studies should investigate the potential impact of socio‐economic factors and professional experience, as they may affect the preferences as well as patterns of work schedules of per‐diem nurses. Moreover, the characteristics and distribution of organisations making bookings on the platform could have an impact on the work schedules because they may have specific demands for per‐diem nurses with respect to volume, shift types and nurse‐related qualifications.

The analyses do not fully reflect the dynamic process on the platform as a marketplace. Future research studies should focus on the adaptive reciprocal behaviour of per‐diem nurses and health institutions over the active periods and the determinants of matchmaking on the platform. Longitudinal analyses should be conducted to investigate whether nurses adjust their availability strategies based on prior booking success or failure. Pre‐planning preferences may also be affected by past conversion rates, which may result in adaptive behaviour on the platform, leading per‐diem nurses to offer shift types with a higher probability of being booked by institutions. This could be determined by analysing changes in availability patterns of per‐diem nurses over time, accounting for the success rates of the availabilities. Lastly, the results must be interpreted in consideration of the nursing shortage during the period of analysis. Studies with longitudinal data could account for the actual situation on the nurse labour market.

## 6 Conclusions

This study is the first to analyse data from online labour platforms to investigate the work preferences and work schedules of per‐diem nurses. This makes a contribution to the current research, which is based primarily on data from surveys and qualitative interviews. The study clearly shows that with the emergence of online platforms, new data sources are becoming available for analysing the extent, patterns and characteristics of per‐diem nurse work. Furthermore, the study results contribute to a greater understanding of the work preferences of per‐diem nurses based on binding shift availability offers and shift work characteristics documented by the bookings. This will inform future discussions about the deployment of per‐diem nurses in shift scheduling systems and the consequences for institution‐related and nurse‐related outcomes. The results show that per‐diem nurses pre‐plan their shift availabilities monthly, and these are booked at a high match rate by the institutions. Institutions book per‐diem nurses in advance rather than for short‐term demand for the various shift types. The advance booking of per‐diem nurses and their use as supplemental staff highlight how Swiss healthcare institutions leverage labour market flexibility to manage workforce demands. The data indicate that this temporary work is more of a side job than a substitute for a permanent position, due to the low number of shift assignments and bookings in a single‐digit number of institutions. The predominance of per‐diem work reflects a broader trend within the gig economy, where platform‐based employment tends to supplement rather than replace traditional jobs (Kässi and Lehdonvirta 2018). Although per‐diem nurses can be situated within the secondary labour market, their working conditions in the study exhibit only limited aspects of the insecurity and marginalisation typically associated with such positions, as described by Dual Labor Market Theory (Doeringer and Piore 1985).

The generalisability of the study findings is limited to per‐diem nurse work in single shifts. Longer periods of employment of a temporary nurse may present different work preferences and booking characteristics. Further, the findings depend on the characteristics of the online labour platform, which in this study are as follows: (1) An agency operates the platform, recruits the per‐diem nurses and makes contracts with the institutions. (2) On the platform, shift availabilities placed by per‐diem nurses can be booked by institutions on a first come, first served basis. There are other online labour platforms for per‐diem nurse work that use different booking mechanisms; for example, some platforms allow institutions to post vacant shift offers that can then be accepted by the per‐diem nurses, enabling the demand side to determine the market and putting the nurses in the decision‐making role. On the platform in the study, per‐diem nurses can exclude institutions in which they do not want to work, in which case their shift availabilities will not be visible to those institutions. Moreover, the market dynamics of online platforms are determined by the actual labour market conditions. The platform data of the study reflect a Swiss nurse labour market in 2022 characterised by a shortage of nurses, with nurses leaving permanent jobs to work instead as temporary nurses.

### 6.1 Implications for Healthcare Management

Online labour platforms for per‐diem nurse work facilitate the booking and management of different forms of flexible nurse work arrangements, such as per‐diem nurse work, as well as intra‐organisational and inter‐organisational float pools (Ahmadi Shad et al. 2025). The data from these platforms reveal the characteristics and patterns of shift availabilities and shift demands across units over time, and this can be considered in planning prospective shifts. Taking the particular characteristics and setting of this study into account, the findings suggest the following: per‐diem nurse work can either be used for applying a mix of permanent and flexible nursing staff to account for variations in bed occupancy rates, or to use temporary nurses to cover short‐term absences due to illness. The broad variety of shift type availabilities and the observed monthly pre‐bookings on the platform indicate that shift managers can consider using per‐diem nurses both in prospective shift planning for expected shift demands and on a short‐term basis as replacements for absent permanent nurses. However, the potential for short‐term replacement by per‐diem nurses is called into question by the monthly pre‐planning time of per‐diem nurses and the immediate pre‐booking by institutions some days after per‐diem nurses have offered a shift availability because there may not be any nurses available for a short‐term assignment. However, the prospective shift planning process could be supported by forecasts based on platform data or per‐diem nurse availability from past planning periods (Kaw et al. 2020). The high match rates and early pre‐bookings on the platform reveal a high demand for per‐diem nurse work in the Swiss healthcare market, which reflects the nursing shortage in the health system. Nursing managers must incorporate the scarcity of temporary nurses in their booking behaviour.

The low number of shifts worked as a per‐diem nurse per institution and the number of different institutions in which per‐diem nurses work confirm the challenges of low institution‐related work experience of per‐diem nurses. This could be overcome by using internal float pools (Seo and Spetz 2013) or booking the same external per‐diem nurses more frequently.

The low shift availability annually of most of the per‐diem nurses indicates that these nurses are not working through the platform as a replacement for a permanent position but rather working some shifts alongside their main employment or during a professional break. Therefore, institutions should view per‐diem nurse work less as a competing work model for permanent positions and more as an alternative work model for nurses who might otherwise leave the profession and, along with this, as an optional resource pool for shift staffing.

## Author Contributions

**Florian Liberatore:** conceptualisation, writing – original draft preparation and funding acquisition. **Marcel Dettling:** methodology/statistics, formal analysis and investigation. **Florian Liberatore, Marcel Dettling:** writing – review and editing and contributed to the study conception and design.

## Funding

This research as a part of the CroWiS project was supported by the Swiss National Science Foundation through the National Research Program ‘Digital Transformation’ (NRP 77; grant 187433).

## Disclosure

Data collection statement: Data utilised in the submitted manuscript have been lawfully acquired in accordance with the Nagoya Protocol on Access to Genetic Resources and the Fair and Equitable Sharing of Benefits arising from their utilisation to the Convention on Biological Diversity.

Statement about statistics: The authors have checked to make sure that our submission conforms as applicable to the Journal's statistical guidelines. There is a statistician on the author team (Marcel Dettling). The authors affirm that the methods used in the data analyses are suitably applied to their data within their study design and context, and the statistical findings have been implemented and interpreted correctly. The authors agree to take responsibility for ensuring that the choice of statistical approach is appropriate and is conducted and interpreted correctly as a condition to submit to the Journal.

## Ethics Statement

The study was submitted to the Cantonal Ethics Committee of the Canton of Zurich, Switzerland. The study does not fall under the Human Research Act and an exemption of an ethical review was received (BASEC‐Nr. Req‐2022‐00708). The usage of the temporary nurse data of the platform was based on an informed consent with opt‐out system. All employees of the temporary agency were informed by a letter of information about the study and could dissent the use of their individual data. The employee ID as identifier of the entries in the platform data was replaced by an alphanumeric code by the temporary agency to guarantee confidentiality. Platform data were stored in a server protected by a password and accessible only to the members of the research team.

## Consent

The usage of the temporary nurse data of the platform was based on informed consent with opt‐out system. All employees of the temporary agency were informed by a letter of information about the study and could dissent the use of their individual data.

## Conflicts of Interest

The authors declare no conflicts of interest.

## Data Availability

The data that support the findings of this study are available from Careanesth AG. Restrictions apply to the availability of these data, which were used under licence for this study. Data are available from the author(s) with the permission of Careanesth AG.
